# Efficient Synthesis of Chlorin e6 and Its Potential Photodynamic Immunotherapy in Mouse Melanoma by the Abscopal Effect

**DOI:** 10.3390/ijms24043901

**Published:** 2023-02-15

**Authors:** Rajeev Shrestha, Shyam Kumar Mallik, Junmo Lim, Pallavi Gurung, Til Bahadur Thapa Magar, Yong-Wan Kim

**Affiliations:** Dongsung Cancer Center, Dongsung Biopharmaceutical, Daegu 41061, Republic of Korea

**Keywords:** Chlorin e6, photodynamic therapy, photosensitizer, antitumor activity, immune response

## Abstract

Photodynamic therapy (PDT) can eradicate not only cancer cells but also stimulate an antitumor immune response. Herein, we describe two efficient synthetic methodologies for the preparation of Chlorin e6 (Ce6) from *Spirulina platensis* and address the phototoxic effect of Ce6 in vitro along with antitumor activity in vivo. Melanoma B16F10 cells were seeded and phototoxicity was monitored by the MTT assay. The C57BL/6 mice were subcutaneously inoculated on the left and right flank with B16F10 cells. The mice were intravenously injected with Ce6 of 2.5 mg/kg and then exposed to red light (660 nm) on the left flank tumors 3 h after the injection. The immune response was studied by analyzing Interferon-gamma (IFN-γ), tumor necrosis factor-alpha (TNF-α), and Interleukin-2 (IL-2) of the right flank tumors through qPCR. Our results revealed that the tumor was suppressed not only in the left flank but also in the right flank, where no PDT was given. The upregulated gene and protein expression of IFN-γ, TNF-α, and IL-2 revealed antitumor immunity due to Ce6-PDT. The findings of this study suggest an efficient methodology of Ce6 preparation and the efficacy of Ce6-PDT as a promising antitumor immune response.

## 1. Introduction

Melanoma is an extremely aggressive malignant tumor that originates from melanocytes, and its advancement is difficult to forecast as it can also develop in other tissues such as the eyes, nasal cavity, anal canal, digestive tract, and genitourinary tract [1,2]. Despite the current guideline-based therapies for patients with melanoma including surgery, radiotherapy, chemotherapy, immunotherapy, and targeted therapy [3], PDT has been successfully used to treat patients having non-melanoma skin cancer [4]. In recent years, PDT has evolved as a promising alternative to the existing therapeutic modality for the treatment of various cancers due to its less invasiveness and minimal systemic toxicity [5]. PDT comprises the use of photosensitizers (PSs) and appropriate light (600–800 nm) along with molecular oxygen to generate reactive oxygen species (ROS) or singlet oxygen (^1^O_2_), which are responsible for cancerous cell death by apoptosis, necrosis, or destruction of blood vessels [6]. PDT has the ability to trigger a localized immune response that fights against cancers, but it also has a systemic abscopal effect that promotes the regression of metastatic and distant contralateral malignancies [7,8]. An increase in antigen presentation following a higher ratio of immunologic cell death (ICD) within the tumor microenvironment can enhance antitumor immunity via the release of primary cytokines such as IFN-γ, TNF-α, and IL-2 followed by activation of dendritic cells (DCs), triggering CDLs to stimulate CD4+ and CD8+ T cells and further CTL (cytotoxic T lymphocyte) and natural killer (NK) cell proliferation [9,10].

Since the PSs have played a vital role in PDT activities, various photosensitizers have been developed, mostly belonging to the tetrapyrrole structure, that have an absorbance wavelength between 600 and 800 nm, exhibit no dark toxicity, and have relatively rapid clearance from normal tissues after PDT, thereby minimizing phototoxic side effects [11]. Therefore, the development of PSs with a highly selective affinity towards tumor-killing activities is always demanded. In this regard, Ce6 has been developed as a promising second-generation photosensitizer due to not only its higher phototoxic potential but also its strong absorption in the red region of the visible spectrum, which leads to penetrating deeper cancer tissue layers [12]. Thus, Ce6-PDT demonstrates significant clinical achievement in the treatment of various cancers, including pancreatic [13], melanoma [14], bladder [15], and breast cancers [16].

**Scheme 1:**
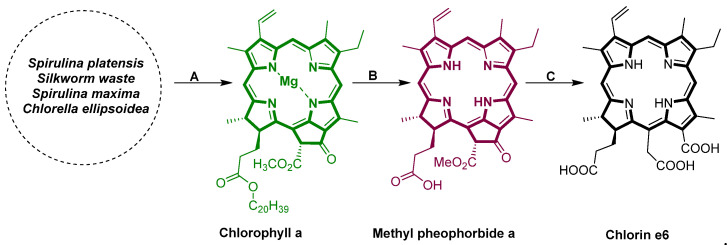
Synthesis of Chlorin e6 via methyl pheophorbide. (**A**) Extraction of chlorophyll a. (**B**) Conversion to methyl pheophorbide a from chlorophyll a. (**C**) Conversion to Ce6.

Considering the therapeutic efficiency, researchers have explored various methods to synthesize Ce6. Notably, Ce6 is prepared from chlorophyll a. The extraction of chlorophyll a can be achieved from different algae or plants, including *Erythrina variegate* [17], bamboo leaves [18], *Erythrina oricntalis* leaves, mulberry leaves, *Pennisetum purpureum*, *Chlorella ellipsoidea* [19], etc. In this regard, Zhang’s group extracted crude chlorophyll from crude silkworm excrement and prepared Ce6 through reverse Claisen condensation, hydrolysis, and acidification [20]. In addition, the accumulated findings suggest that Ce6 was prepared by either methyl pheophorbide a (Scheme 1) [21,22,23] or pheophytin a [24,25], which was converted from chlorophyll a. However, the method of preparing pheophytin a is much easier and the reaction is simpler to handle.

In the course of developing an efficient method to prepare Ce6, we followed two methods via pheophytin a from chlorophyll a, which is extracted from *Spirulina platensis*. The methods were modified in various ways by altering the quantity of solvents, extraction or reaction duration, and chemical reagents. Therefore, our first aim was to find the best methodological strategy to prepare Ce6 and analyze its PDT in vitro by using melanoma B16F10 cells. We also studied the immune response in vivo by irradiating light into the left flank of mice, inducing the regression of untreated right flank tumors. In addition, gene and protein expression of the primary cytokines such as IFN-γ, TNF-α, and IL-2 were also examined to support the antitumor immunity by Ce6-PDT.

## 2. Results

### 2.1. Synthesis of Ce6 

Ce6 was prepared through two different methodological strategies. Method 1 and method 2 were analyzed and compared and are summarized in Table 1. Method 1 involved the extraction of chlorophyll a for 12 h by using 99.9% EtOH, whereas in method 2, 96% EtOH and hexane were used for 12 h. Although the same solvent, hexane, was used in both methods for the synthesis of pheophytin a from chlorophyll a, the addition of double-strength HCl (2 N) in method 2 reduced the reaction time from 4 h to 2 h. When 13% ethanolic solution of KOH was used in acetone reflux of pheophytin a in method 2, the reaction was completed in 10 min, but in method 1, it took 12 h for the completion of the reaction by using 1 M NaOH. After obtaining Ce6, the purification process was also performed in two different ways. Method 1 included a series of steps, i.e., pH change of Ce6 solution to 7, centrifugation of solution followed by filtration of the supernatant, pH change of the Ce6 solution to ~3.5, and filtration. Similarly, method 2 included pH alteration of Ce6 solution to 0.6, filtration, pH change of filtered Ce6 solution to ~3, and, again, filtration. The consumption of 11% *v*/*v* HCl and 30% NaOH was greater during the purification process in method 2 than in method 1. Among the two methods, the preparation time duration of Ce6 was reduced in method 2 more than in method 1. The final yield was similar, but the purity of Ce6 in method 1 was slightly improved over that of method 2. The formed Ce6 with PVP (1:1) is coined as Phonozen^®^, Dongsung Biopharmaceutical, Seoul, Republic of Korea.

### 2.2. NMR, HPLC, and LC/MS Analysis

**Figure 1:**
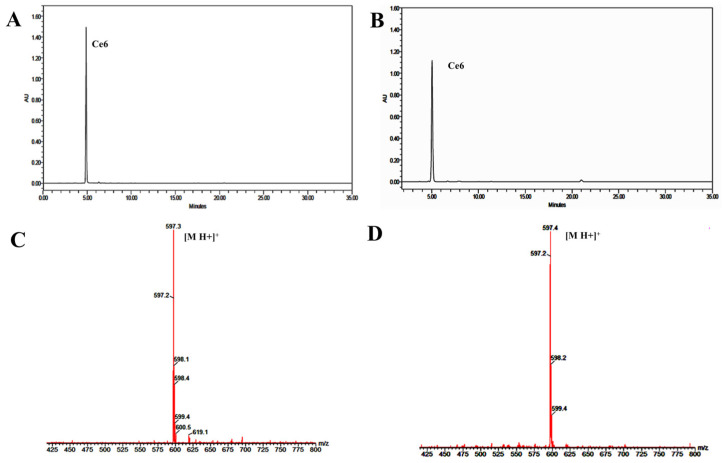
HPLC chromatogram and mass spectrogram of Ce6 by method 1 and method 2. Separation condition is linear gradient of 45–100% (acetonitrile) in 0.1% TFA water in a reverse-phase HPLC column. (**A**) HPLC chromatogram of Ce6 by method 1. (**B**) HPLC chromatogram of Ce6 by method 2. (**C**) LC/MS of Ce6 by method 1. (**D**) LC/MS of Ce6 by method 2.

The synthesized Ce6 from two different methods was analyzed and compared through NMR, HPLC, and LC/MS. The HPLC analysis of Ce6 revealed an RT of 4.9 at 407 nm (Figure 1A,B). Similarly, the two compounds were also confirmed by LC/MS H^+^ as 597 (Figure 1C,D). In addition, during the reaction, chlorophyll a and pheophytin a were analyzed and compared through HPLC. They were detected at 5.1 and 9.53 RT at 430 nm (Figures S1, S2, S4, and S5), respectively. From the ^1^H NMR analysis of Ce6 by two methods, both revealed similar peak values at the same chemical shifts, including –OH peaks of three carboxylic acids at *δ* 9.75 (s), 9.65 (s), and 9.10 (s) of method 1 and *δ* 9.73 (s), 9.61 (s), and 9.10 (s) of method 2 (Figures S3 and S6). Ce6 obtained from methods 1 and 2 was found to be 97% and 94% pure, respectively. 

### 2.3. PH Selection of Ce6 through UV and Fluorescence

**Figure 2:**
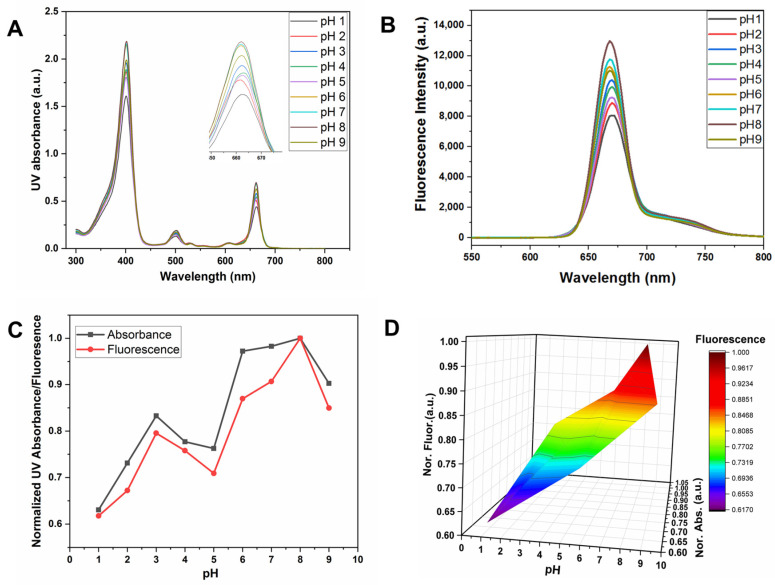
Effect of different pH (1–9) of Ce6 in UV absorbance and fluorescence. (**A**) UV absorption spectra of Ce6 (pH 1–9). (**B**) Fluorescence intensity of Ce6 (pH 1–9). (**C**) Comparison of normalized absorbance (Nor. Abs.) and fluorescence intensity (Nor. Fluor.). (**D**) Corresponding 3D plot. The Ce6 was analyzed in 10 ppm. Absorbance and fluorescence wavelengths were at 662 and 668 nm, respectively.

**Figure 3:**
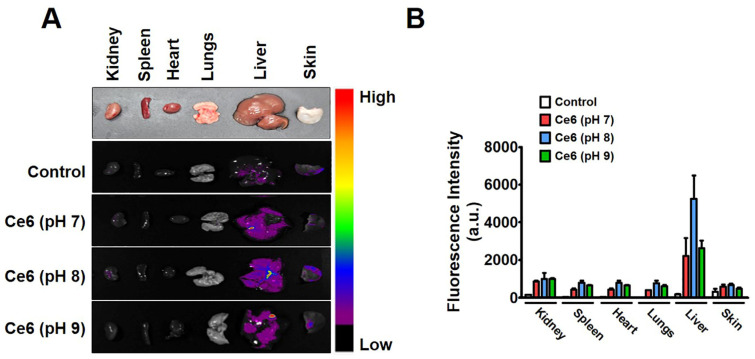
In vivo biodistribution of Ce6 (pH 7, 8, and 9) treatment groups in C57BL/6 mice. (**A**). Ex vivo fluorescence images of the dissected organs (kidney, spleen, heart, lungs, liver, and skin) 2 h post injection of Ce6 solutions at pH 7, 8, and 9 in C57BL/6 mice. (**B**). Quantitative signal analysis of organs (kidney, spleen, heart, lungs, liver, and skin). Data were presented as mean ± SD (*n = 3*).

Dihydroporphyrin (chlorin) compounds exhibit absorption maxima of the two most intense bands, which are located in the visible range near 402 ± 4 nm (Soret band) and 660 ± 5 nm (Q-band) [26]. In order to select the suitable pH of Ce6 for the in vitro and in vivo experiment, the absorbance and fluorescence of Ce6 (10 ppm) were analyzed and compared at different pH, ranging from pH 1 to 9 (Figure 2A–D). The results showed that the maximum absorbance and fluorescence of Ce6 were observed at pH 8. After having this result, we further investigated the biodistribution of Ce6 by injecting Ce6 solutions at pH 7, 8, and 9 in C57BL/6 mice. Mice were sacrificed for the collection of tissues from different organs after 2 h post injection. The ex vivo fluorescence imaging of the major organs indicated a high accumulation of Ce6 at pH 8 (Figure 3A). To further examine the biodistribution of Ce6 (pH 7, 8, and 9) in different organs, Ce6 was extracted from the organ and determined by fluorescent measurement. Both fluorescence imaging and fluorescent measurement results showed that Ce6 at pH 8 could concentrate more predominately in various mice tissues as compared to Ce6 at pH 7 and 9. The result demonstrated that the fluorescence intensity in liver tissue was higher than that in the lungs, spleen, kidney, heart, and skin. The biodistribution of the different Ce6 at pH 7, 8, and 9 in each organ at 2 h post injection and its representation bar are plotted in Figure 3B. Moreover, Ce6 demonstrated rapid elimination patterns from normal tissue, with less fluorescence signal after 3 h (SI-7, Figures S7 and S8). 

### 2.4. In Vitro Cytotoxicity of Ce6-PDT in Melanoma Cancer

**Figure 4:**
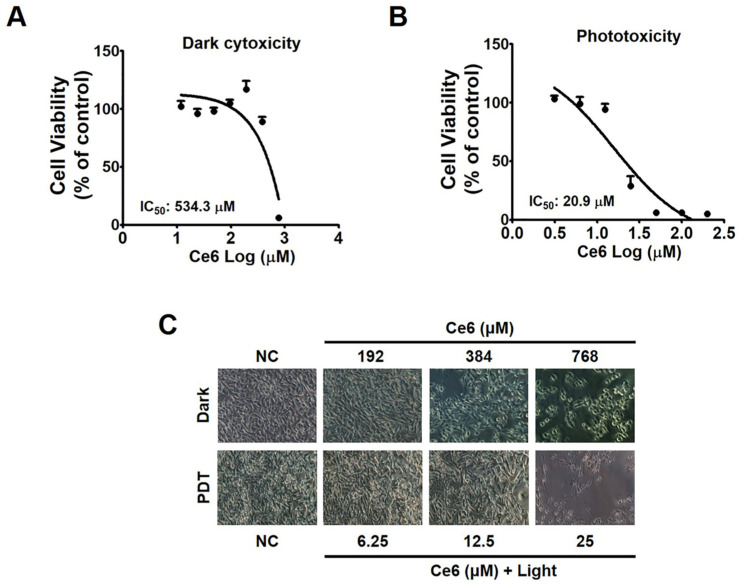
Ce6-mediated cytotoxicity on the melanoma cancer cells by the MTT assay. (**A**) Cytotoxic activity of Ce6 against B16F10 cells in the dark compared with vehicle-treated control. (**B**) Cytotoxic activity of Ce6-PDT against B16F10 cells compared with vehicle-treated control. (**C**) Morphological changes in B16F10 cells with or without Ce6 (192 µM, 384 µM, and 768 µM) in the dark and with or without Ce6 (6.25 µM, 12.5 µM, and 25 µM) followed by 660 nm laser at 50 mW for 200 s (1 J/cm^2^) in phototoxicity. The data are the presentation of mean ± SEM obtained from three independent experiments.

To investigate the cytotoxic effect of Ce6 under laser irradiation against melanoma cancer cells (B16F10), the MTT assay was performed (Figure 4A–C). Ce6 in the dark showed the highest cytotoxicity at a concentration of 768 µM (IC50 = 534.3 μΜ) (Figure 4A), whereas, at the concentration of 25 µM, the combination of Ce6 and 660 nm laser irradiation at 50 mW for 200 s (1 J/cm^2^) induced phototoxicity in B16F10 cells (IC50 = 20.98 μΜ) in comparison to vehicle-treated control (Figure 4B). Control was treated with only normal saline. As a result, the finding suggests that Ce6 in the dark has minimal toxicity, which is good for the ideal photosensitizer. On the other hand, Ce6-PDT demonstrated high cytotoxicity. 

### 2.5. In Vivo Effect of Ce6-PDT in Allograft Mouse Model 

**Figure 5:**
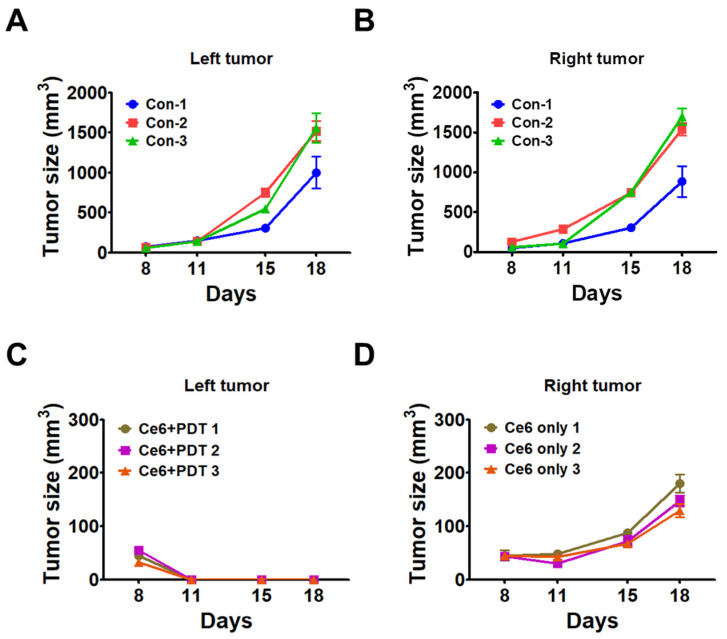
Tumor volume changes in control and Ce6-PDT groups with tumors in the left and right flanks. The mice were subcutaneously injected at the left and right flank with 0.1 mL of B16F10 cells (1 × 10^6^ cells/mL). The vehicle control was injected with normal saline. (**A**) Changes in volume of the left tumor in the control group. (**B**) Changes in volume of the right tumor in the control group. (**C**) Changes in volume of irradiated left tumor in Ce6-PDT group. (**D**) Volume changes of the non-irradiated right tumor in the Ce6-PDT group. The data are the representation of mean ± SEM (*n = 3*).

Since the Ce6-PDT demonstrated phototoxicity in vitro, we used an allograft mouse model using B16F10 melanoma cells to investigate Ce6 efficacy as a PDT agent. In this regard, C57BL/6 mice were transplanted with B16F10 cells for the tumor allograft model. Tumor-bearing mice in the vehicle control and Ce6-PDT groups were injected intravenously with vehicle (normal saline) and 2.5 mg/kg Ce6 solution via the tail vein, respectively. As shown in Figure 5A,B, the tumor volume increased after the subcutaneous transplantation in the control group. As shown in Figure 5C, after Ce6-PDT treatment on the left flank, the tumor volume was considerably decreased. On the other hand, the growth of the right tumor was also suppressed without Ce6-PDT treatment on the right flank (Figure 5D) compared to the control group on day 11, but it then resumed growing until the 18th day. Suppressed tumor growth of the right flank was due to the immune responses generated by Ce6-PDT treatment on the left flank.

### 2.6. Cytokine Response after Ce6-PDT

**Figure 6:**
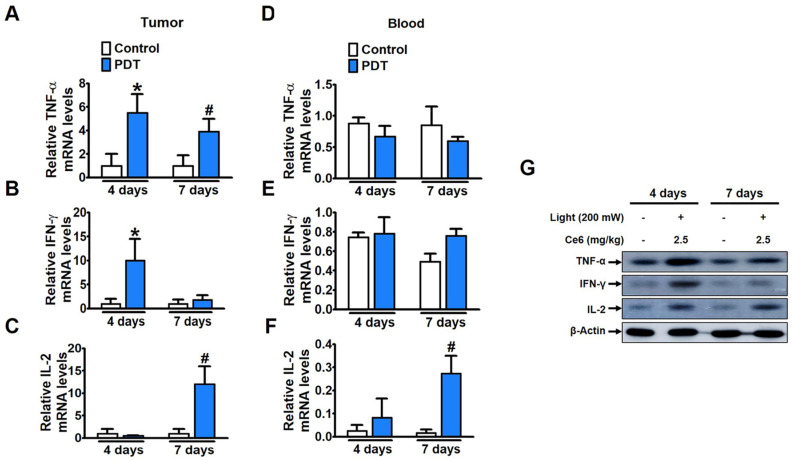
Th1-related cytokine expression level changes after Ce6- PDT treatment in B16F10 melanoma mice tumor. Real-time PCR expression of (**A**) TNF-α, (**B**). IFN-γ, and (**C**) IL-2 level in the non-irradiated right tumors and (**D**–**F**) in serum on the 4th and 7th day after Ce6-PDT. (**G**) Protein expression of TNF-α, IFN-γ, and IL-2 levels in the non-irradiated right tumors on the 4th and 7th day after Ce6-PDT treatment. Values are shown as mean ± SEM obtained from at least three independent experiments and were analyzed by one-way ANOVA with Tukey’s post hoc test. * *p* < 0.05 compared with the control group in 4 days. ^#^ *p* < 0.05, compared with the control group in 7 days.

We aimed to identify the immune response involved in PDT’s antitumor effect since we discovered that the non-irradiated right tumor also showed reduced growth along with the irradiated left tumor. Real-time PCR analysis was performed on the tumor tissues of non-irradiated tumors in the right flank. When compared to the control in the right non-irradiated tumor, TNF-α expression was found to increase significantly on the fourth and seventh days after PDT treatment of the left tumor (Figure 6A). In the case of IFN-γ, its expression was observed to be considerably higher on the fourth day compared to that on the seventh day of Ce6-PDT treatment (Figure 6B). On the other hand, upregulation of IL-2 on the seventh day was more than on the fourth day of treatment (Figure 6C). These findings imply that Ce6-PDT induced systemic effects in non-irradiated tumors by triggering an immune response. We further confirmed the systemic effects of Ce6-PDT by using blood serum (10 µL) through mRNA expressions of cytokines. TNF-α and IFN-γ expression levels remained unchanged and were comparable to control levels on the fourth and seventh days, but IL-2 levels increased greatly on the seventh day after Ce6-PDT therapy, mimicking the pattern as seen in the tumor (Figure 6D–F). We also obtained similar changes in the protein expression of TNF-α, IFN-γ, and IL-2 in the right non-irradiated tumor via Western blot (Figure 6G) as seen in the PCR analysis. According to previous and current findings, cytokines including TNF-α, IFN-γ, and IL-2 are powerful inducers of antitumor immunity [27]. Therefore, the results here indicate that, due to the elevated expression of TNF-α, IFN-γ, and IL-2, the growth of the non-irradiated right flank tumors was decelerated.

## 3. Discussion

Ce6 is a second-generation photosensitizer that is predominantly utilized for the treatment of various cancers, including melanoma [28]. Melanoma is a destructive form of skin cancer that has a very low rate of patient survival due to resistance to most therapeutic strategies [1]. Therefore, in order to assess the anticancer effectiveness of Ce6- PDT both in vitro and in vivo utilizing a melanoma mouse model, this study concentrated on the efficient synthesis of Ce6 with high yield and purity. For the development of Ce6, we have studied two methodological strategies for the synthesis of Ce6, analyzed each step, and compared them with each other. When comparing the two methods of preparing Ce6, method 1 was introduced as the superior strategical protocol as revealed by its 97% purity and simplicity in handling the reaction. However, method 1 took longer than method 2 in the overall steps. Although Ce6 absorption in the red light region has been significantly demonstrated [29], the current study tested Ce6 absorbance and fluorescence in various pH ranges (pH 1–9). We found that Ce6 exhibited the highest absorbance and fluorescence intensity at pH 8. Further, we found that the maximum fluorescence intensity for Ce6 absorbance was at pH 8 in the kidney, spleen, heart, lungs, liver, and skin of C57BL/6 mice while the highest biodistribution for all Ce6 at pH 7, 8, and 9 was in the liver tissues. 

Ce6-PVP has shown a short half-life (t_1/2_) in normal tissues than in tumor tissue and, most importantly, it is cleared from the organ within 24 h, which has the advantage of decreasing drug accumulation and thus reducing toxicity [30,31,32]. Our pharmacokinetics result for Ce6 is in accordance with the above-cited results. Ideal PSs show minimal toxicity in the absence of light and are only cytotoxic in the presence of light at a defined wavelength [33]. To achieve an optimal cytotoxic response of Ce6 and its PDT in melanoma cancer, B16F10 cells were studied. For the PDT, the melanoma cells were exposed to Ce6 (0–768 μM) in the dark and Ce6 (0–100 μM) with irradiation at 660 nm (50 mW, 1 J/cm^2^) for 200 s. Notably, Ce6 caused a significant PDT response at the concentration of 25 µM with an IC50 of 20.98 μΜ. The result also demonstrated the lower toxicity of Ce6 in the absence of light with an IC50 of 534.3 μΜ, which is a beneficial factor for PDT. 

Inspired by the encouraging response of Ce6-PDT in vitro, its antitumor effectiveness and abscopal effect were also examined by successfully establishing tumors derived from B16F10 cells in both flanks of syngeneic mice. Only the primary tumors on each mouse’s left side received Ce6-PDT treatment. The tumors on the right side were examined for a potential abscopal impact because they were regarded as distant tumors. Tumors and blood serum were collected on the fourth and seventh days of Ce6-PDT treatment to evaluate the cytokine response. Rapid tumor growth was observed in the control group, but the tumors on the left flank, where the light was irradiated, were significantly diminished, whereas those on the right flank grew slowly. Our findings showed that Ce6-PDT treatment on the left flank of mice led to a restriction in the growth of tumors on the right flank, signifying the antitumor immune responses of Ce6-PDT treatment. Oh and his team demonstrated similar antitumor immune responses by using Ce6-PDT coupled with an anti-CD25 monoclonal antibody [34]. Taken together, Ce6-PDT had an abscopal effect in addition to having a direct effect on melanoma tumor growth.

PDT stimulates the immune system through a variety of mechanisms, including the release of previously concealed tumor-associated antigens (TAAs) and immune-stimulatory molecules from tumors, which could activate and prompt an anticancer immune response. In addition, PDT also encourages chemokine and cytokine release to produce systemic influence and further activate the immune system’s antitumor activities [35]. An earlier study discovered that combining TNF-α and IFN-γ causes inflammatory cancer cell death via PANoptosis as well as an adaptive immune response on the levels of their increment [36,37]. Moreover, the production of IL-2 contributes to immune stimulation by leading to rapid lymphocyte proliferation and amplification of antigen-specific responses [38]. 

Therefore, for additional investigation of the antitumor immune response of Ce6-PDT, we analyzed the protein and mRNA levels of Th1-related cytokines such as TNF-α, IFN-γ, and IL-2 in non-irradiated tumors of Ce6-PDT groups. TNF-α was significantly increased on the fourth and seventh days of Ce6-PDT treatment compared to the vehicle control at the gene and protein levels. In addition, on the fourth day, the mRNA and protein expression of IL-2 was slightly downregulated, but on the seventh day, it was four times higher than that of the control. The expression of IFN-γ was noticeably higher on the fourth day compared to the seventh day of PDT treatment. While serum TNF-α and IFN-γ levels did not change, IL-2 levels were upregulated similarly to those seen in melanoma tissues on the fourth and seventh days after treatment with Ce6-PDT. This evidence implies that Ce6-PDT boosted the immune response by increasing TNF-α, IFN-γ, and IL-2 cytokine to produce systemic effects in the non-irradiated tumor. Our findings are also consistent with previous reports that pyrolipid nanoparticle-PDT contributed to the enhancement of antitumor immunity by elevation of TNF-α, IFN-γ, and IL-2 on the first day after PDT treatment but cytokine levels were found to decrease on the second day [39]. According to previous studies, Ce6 associated with hybrid protein oxygen nanocarrier [40] and incorporated with dissolving microneedle-mediated PDT [41] showed a significant abscopal effect elicited by activating ICD, which in turn promoted DC’s maturation and the subsequent antigen presentation, thereby facilitating the T-cell-mediated immune response. However, for the first time, we have reported the abscopal effect of Ce6-PDT without capsuling any nanoparticles and not in combination with other therapies.

## 4. Materials and Methods

### 4.1. Synthesis of Ce6

**Scheme 2:**
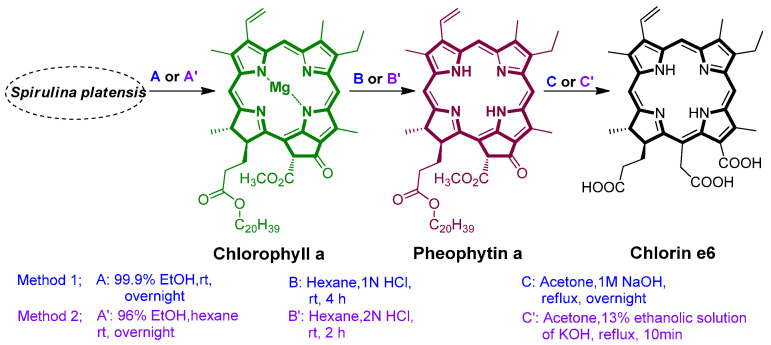
Extraction of chlorophyll a and synthesis of pheophytin a and Ce6 through method 1 and method 2.

We developed two new methods, i.e., method 1 and method 2, which included three steps: (a) extraction of chlorophyll a from *Spirulina* powder, (b) demetallation of chlorophyll a to convert it into pheophytin a, and (c) conversion to Ce6 from pheophytin a. These methods were accomplished by altering the reaction procedure as well as different conditions with each other (Scheme 2).

#### 4.1.1. Method 1. Chlorophyll a Extraction and Synthesis of Ce 6

- Extraction of chlorophyll a

Spirulina (200 g) was dispersed in EtOH (2L) and stirred overnight (12 h) in a continuous nitrogen environment. The *Spirulina* was filtered, and the filtered ethanol solution was evaporated through a vacuum evaporator until the volume of ethanol remained at 400 mL. Then, water (200 mL) and hexane (500 mL) were added to the chlorophyll a-containing EtOH solution, stirred for 10 min, and kept in the refrigerator overnight. Later, chlorophyll a was extracted in hexane by liquid–liquid layer separation three times.

- Conversion to pheophytin a

To the hexane solution of chlorophyll a, 1 N HCl (7 mL) was added dropwise and stirred for 4 h at room temperature. Then, 1 M NaOH (10 mL) was added dropwise after the completion of the reaction analyzed by TLC. Then, 70% EtOH (400 mL) was added to the reaction mixture, which was again kept in the refrigerator for 12 h. The hexane layer was separated by liquid–liquid separation with 70% EtOH three times, vacuum evaporated, and then dried to obtain pheophytin a.

- Conversion and purification of Ce6

Pheophytin a (4.92 g) was dissolved in acetone (400 mL) and nitrogen bubbled into the solution of pheophytin a for 30 min. Then, 1 M NaOH (20 mL) was added dropwise and refluxed overnight under inert conditions. After completion of the reaction monitored by TLC, the reaction mixture (Ce6) was filtered and washed with acetone. The black solid of Ce6 was collected and dried for 4 h. The dried Ce6 (3.92 g) was dissolved in 25 mL of water and stirred for 2 h at room temperature under inert conditions. Then, 1 N HCl (7.5 mL) was added to the solution to maintain pH 7.0 and stirred for 2 h. The reaction mixture was centrifuged at 10,000 rpm for 1.5 h to remove junk such as carbohydrates, peptides, and others. The supernatant was filtered, then the filtrate was treated with 1 N HCl to maintain pH ~3.5, and Ce6 was collected through filtration. It was vacuum dried at 35 °C for 4 h to obtain a 1.75 g yield (1% of *Spirulina* powder). Ce6 was analyzed through NMR and LC/MS. ^1^H-NMR (600 MHz, DMSO-d_6_); *δ* 9.75 (s, 1H), 9.65 (s, 1H), 9.10 (s, 1H), 8.23 (dd, 1H, *J* = 18, 18 Hz), 6.41 (dd, 1H, *J* = 18, 18 Hz), 6.13 (dd, 1H, *J* = 18, 18 Hz), 5.37–5.41 (q, 2H, *J* = 18 Hz), 4.61–4.65 (q, 1H, *J* = 6 Hz), 4.45–4.47 (q, 1H, *J* = 6 Hz), 3.71–3.75 (q, 2H, *J* = 6, 12 Hz), 3.59 (s, 3H), 3.49 (s, 3H), 3.25 (s, 3H), 2.63–2.68 (m, 1H), 2.29–2.34 (m, 1H), 2.14–2.19 (m, 1H), 1.68 (d, 6H, *J* = 6 Hz), 1.65 (t, 1H, *J* = 6, 12 Hz). Mass spectra m/z C_34_H_36_N_4_O_6_ (M+H)^+^: 597. Yield: 1.75 g, <1% of *Spirulina* powder.

#### 4.1.2. Method 2. Chlorophyll a Extraction and Synthesis of Ce6

- Extraction of chlorophyll a

Spirulina (100 g) was dispersed in hexane (200 mL) for 30 min, 96% EtOH (800 mL) was added, and it was stirred overnight (12 h) in a continuous flow of nitrogen. Then, spirulina was filtered, water (200 mL) was added to the filtrate, and the hexane layer was separated through the liquid–liquid separation method to obtain chlorophyll a.

- Conversion to pheophytin a

To the hexane solution of chlorophyll a, 5 mL of 2 N HCl was added dropwise and stirred for 2 h. After completion of the reaction checked by TLC, the reaction mixture was washed through liquid–liquid separation by using 80% EtOH (200 mL) twice. Pheophytin a in the hexane layer was collected, vacuum evaporated, and dried for an hour.

- Conversion and purification of Ce6

Pheophytin a (2.5 g) was dissolved in acetone (60 mL) and nitrogen bubbled into the solution of pheophytin a for 30 min. Then, 13% KOH in ethanol (378 mL) was added dropwise and refluxed for 10 min. After the completion of the reaction checked by TLC, the reaction mixture was cooled down to 17 °C and kept in an ice bath. Then, 11% *v*/*v* HCl was added to the reaction mixture to maintain pH 0.2 and stirred for an hour for acidification. Then, 30% NaOH solution was added to the solution to maintain pH 0.6 and stirred for 30 min for Ce6 cleaning while maintaining a temperature of 17–19 °C. The reaction solution was filtered, where almost all the junk such as carbohydrates, peptides, carotenoids, and others was removed. The filtered solution was again treated with the addition of 30% NaOH solution to maintain pH ~3.0 and stirred for an hour. Thus, the formed precipitate of Ce6 was filtered and dried for 24 h at 25 °C. The synthesized Ce6 was analyzed through NMR and LC/MS. ^1^H-NMR (600 MHz, DMSO-d_6_); *δ* 9.73 (s, 1H), 9.61 (s, 1H), 9.10 (s, 1H), 8.21 (dd, 1H, J = 18 Hz, 18 Hz), 6.39 (dd, 1H, J = 18), 6.12 (dd, 1H, J = 18, 18 Hz), 5.37–5.39 (q, 2H, J = 7.2 Hz), 4.61–4.65 (q, 1H, J = 6 Hz), 4.45–4.47 (q, 1H, J = 12 Hz), 3.71–3.74 (q, 2H, J = 6 Hz), 3.59 (s, 3H), 3.48 (s, 3H), 3.22 (s, 3H), 2.63–2.69 (m, 1H), 2.30–2.34 (m, 1H), 2.14–2.19 (m, 1H), 1.68 (d, 6H, J = 6 Hz), 1.63–1.66 (t, 1H, *J* = 6, 12 Hz). Mass spectra m/z C_34_H_36_N_4_O_6_ (M+H)^+^: 597. Yield: 0.8 g, <1% of spirulina powder.

### 4.2. NMR, HPLC, LC/MS, UV, and Fluorescence Analysis

Nuclear magnetic resonance (NMR) was recorded on Bruker 600 MHz for ^1^H NMR. The chemical shift values (*δ*) were recorded in ppm (parts per million). Thin-layer chromatography was performed on silica gel 60 F_254_ (Merck). *Spirulina platensis* was purchased from SHAAN XI FREESUN TRADING CO. LTD, Ningbo, China. Ethanol, acetone, and hexane were purchased from Daejung Chemicals, Siheung-si, Republic of Korea. HPLC-grade water, methanol, and acetonitrile were purchased from Duksan company, Ansan, Republic of Korea. Compounds were analyzed using Waters Alliance separation module e2695 (Waters, Milford, MA, USA), coupled with a 2998 PDA detector (Empower^®^ 3 software). The column used for HPLC was Capcell pak UG120 C18 (4.6 mm × 150 mm, 5 µm) using a linear gradient of 45–100% B (acetonitrile) in 0.1% TFA water (A) over 20 min at a flow rate of 1 mL/min (Ce6). The detection wavelength was set to 407 nm for Ce6 and 430 nm for chlorophyll a and pheophytin a. For chlorophyll a and pheophytin a, isocratic elution of acetonitrile, methanol, and ethyl acetate mixture (3:1:1) was used. The column temperature was set at 23 ± 2 °C. Mass analysis (m/z 200–800) was performed by online HPLC–MS using a Waters ZQ2000 mass detector (Waters, Milford, MA, USA) with ESI positive ionization mode. For ultraviolet–visible (UV) spectrophotometry (Thermo-scientific, Skanlt software 5.0), the samples were prepared in 95% ethanol solvent (Duksan, HPLC grade pure) at a concentration of 10–50 ppm. The data were corrected for solvent background by the instrument’s calibration using 95% ethanol as a blank. The absorption spectra of the sample in solution were obtained in the range of 300–800 nm at a 1 nm interval in three determinations using three trial samples. The fluorescence intensities of Ce6 at different pH were recorded on a Spark^®^ multimode microplate reader (Tecan Trading AG, Männedorf, Switzerland) at the emission wavelength range of 500–800 nm. The samples for fluorescence were prepared at 10–50 ppm in 95% EtOH.

### 4.3. Cell Viability Assay

B16F10 cells were purchased from Korean Cell Line Bank (KCLB, Seoul, Republic of Korea). They were grown in DMEM supplemented with 10% fetal bovine serum (life technologies corporation, Waltham, MA, USA) and 1% Penicillin & Streptomycin (life technologies corporation, Waltham, MA, USA). These cells were cultured at 37 °C in a humidified atmosphere containing 5% CO_2_. B16F10 cells were plated at a density of 5 × 10^3^ cells per well in 96-well plates and then incubated for 3 h. After treatment of the cells with various concentrations of Ce6, the cells were or were not exposed to irradiation of a 660 nm laser with power densities of 1 J/cm^2^ and were further incubated for 72 h. An MTT assay was carried out to determine the cell viability at 590 nm by using a microplate reader.

### 4.4. Biodistribution of Ce6

C57BL/6 mice were injected intravenously with or without Ce6 solutions (2.5 mg/kg) at pH 7, 8, and 9. After the mice were euthanized 2 h post injection, organs (kidney, spleen, heart, lungs, liver, and skin) were collected and weighed. For ex vivo organ distributions of Ce6, samples were placed on a black tray and then subjected to the FOBI imaging system (Neo-Science, Suwon, Republic of Korea). To extract Ce6, tissues were mixed with lysis buffer, homogenized by using a tissue homogenizer, and centrifuged at 9000 rpm for 15 min. The supernatant was collected, and the Ce6 content was measured spectrophotometrically at 660 nm using a Spark Multimode Microplate Reader (Tecan Trading AG, Männedorf, Switzerland). The fluorescence intensity was normalized to the tissue weight.

### 4.5. Animal Model

Six-week-old male C57BL/6 mice (*n* = 10) weighing 21 g were purchased from Orient Bio (Seongnam, South Korea) and the mice were housed in a standard environment (20 ± 2 °C; 50 ± 5% humidity; 12, 12 h light/dark cycle; diet; and filtered water ad libitum) in the animal house facility of Dongsung Cancer Center, Daegu, Republic of Korea for 7 days. Each experimental group consisted of randomly grouped mice of similar weight. All the mouse experiments were reviewed and carried out with the approval of the Institutional Animal Care and Use Committee of the Dongsung Cancer under protocol IACUC #ds002106117-2.

### 4.6. Allograft Mouse Model Using B16F10 Melanoma Cell Line

Ten mice were chosen at random for the study after a week of acclimation according to the previous literature [42]. Mice received a subcutaneous injection of 0.1 mL of B16F10 cells (1 × 10^6^ cells/mL) into both the left and right flank. When the tumor mass reached the range of 50–70 mm^3^ after inoculation, a total of 10 tumor-bearing mice were randomly divided into control (*n* = 5) and Ce6-PDT groups (*n* = 5).

### 4.7. PDT in the Animal Model

The mice were administered saline or 2.5 mg/kg Ce6 intravenously through mouse tail veins in the vehicle control and Ce6-PDT groups after applying anesthesia. After staying in the dark room for 3 h, the mice were irradiated by a red light at a rate of 100 J/cm^2^ for 8 min 20 s. To evaluate the therapeutic efficacy of Ce6-PDT, the tumor volumes of the mice were recorded on days 8, 11, 15, and 18. The tumor volumes (V) were measured by a vernier caliper and were calculated by using the following formula: V = L × W^2^/2, (L: length and W: width). When the tumor reached a size of over 2000 mm^3^, the mice were euthanized by cervical dislocation. Since 2 mice each from the control and Ce6-PDT groups were found to be dead during the experiment, they were not included in the analysis. Only 3 mice from each group were included in the analysis.

### 4.8. Cytokine mRNA Expression Assay

The mRNA expression of IFN-γ, TNF-α, and IL-12 was determined by qPCR. Total RNA was isolated from cells according to the manufacturer’s recommendations using TRIzol reagent and the Absolutely RNA kit from Takara Bio, Kusatsu, Japan. Total RNA (2 μg) was reverse transcribed in a total volume of 20 μL using 200 U of Superscript II reverse transcriptase (Invitrogen, Waltham, MA, USA), 100 pmol oligo-dT, 0.5 mM dNTP, and 40 U RNasin (Promega, Madison, WI, USA). The resulting complementary DNA was diluted 1:10 with nuclease-free water. Five microliters of diluted cDNA was used in subsequent PCR reactions. The PCR amplification protocol was 50 °C for 2 min and 95 °C for 10 min followed the 40 cycles of 95 °C for 30 s, 60 °C for 30 s, and 72 °C for 30 s. The qPCR analysis was performed on a C1000^TM^ Thermal Cycler (CFX 96^TM^ Real-Time System, BioRad, Munich, Germany). All qRT-PCR reactions were carried out in triplicate. Relative quantification with the data obtained was performed according to the user’s manual. The results are presented as transcript levels relative to the levels in untreated control cells, with average mRNA levels of the internal control gene, glyceraldehyde-3-phosphate dehydrogenase, used as the normalization control. To verify changes in gene expression, qPCR was carried out on selected genes. All primers were designed based on nucleotide sequences retrieved from Genbank using the Primer Express software (Applied Biosystems, Foster City, CA, USA). The fold-change (ratio) in gene expression was calculated by using the threshold cycle (CT). The primer pairs used in this study are listed in Table 2.

### 4.9. Western Blot Assay

Briefly, mice were sacrificed by cervical dislocation on the 18th day after tumor inoculation. Total proteins from tumor tissues (20 mg) were extracted using lysis buffer (0.15 M Sodium chloride, 1% Triton X-100, 1% Sodium deoxycholate, 0.1% SDS, 50 mM Tris adjusted to pH 7.5) containing protease and phosphatase inhibitor cocktail (Gen-DEPOT, Katy, TX, USA) and were homogenized by a tissue homogenizer. The tissue lysate was then centrifuged at 13,000× *g* for 15 min, and the protein content in the supernatant was quantitated by using Bio-Rad, a protein assay reagent. The expression of TNF-α, IFN-γ, and IL-2 proteins was determined by Western blot assay. The primary antibodies against TNF-α were obtained from Novus Biologicals, Inc. (Centennial, CO, USA) and antibodies against IFN-γ and IL-2 were obtained from Cell signaling Technology, Inc. (Boston, MA, USA), while antibodies against β-Actin were obtained from Abcam (Cambridge, UK). The proteins from homogenates were separated by polyacrylamide gel electrophoresis and electrophoretically transferred to nitrocellulose membranes. Horseradish peroxidase (HRP)-conjugated anti-IgG antibodies were used as the secondary antibodies. The membranes were washed and incubated for 1 h at room temperature with horseradish peroxidase (HRP)-conjugated anti-IgG antibodies. The membranes were developed using an ECL chemiluminescence reagent (Pierce Biotechnology, Rockford, IL, USA), and were detected on a luminescent image analyzer (ImageQuant Las 500, Cytiva, Tokyo, Japan).

### 4.10. Statistical Analysis

All data were presented as mean ± standard deviation (SD). Data were analyzed using one-way ANOVA followed by Tukey’s post hoc test using GraphPad Prism software (version 5.01, Inc., 2007, San Diego, CA, USA). Statistical significance was accepted at *p* < 0.05.

## 5. Conclusions

Ce6 was successfully synthesized by two new methods, and we compared the methodological strategy, yield, and purity. Among them, method 1 could be a better protocol for the preparation of Ce6 in terms of solvents and laboratory handling; however, the purity and yields of both methods are comparable. Therefore, the subsequent application of Ce6, prepared by method 1, was used for immunological abscopal effect in C57BL/6 tumor mice. Taken together, our results suggest that Ce6-PDT has local antitumor activity, and its immune response, through the production of pro-inflammatory (Th1) cytokines such as TNF-α, IFN-γ, and IL-2, might restrict tumor growth on the non-irradiated right flank of the mice due to the abscopal effect. Therefore, these findings might serve as experimental support for future studies on the anticancer and abscopal effects of Ce6-PDT.

## Figures and Tables

**Table 1:** Comparison of method 1 and method 2.

Parameters	Method 1	Method 2
The solvent used in the extraction	99.9 % EtOH	96% EtOH, hexane
Extraction time duration of chlorophyll a	12 h	12 h
Solvent/acid strength/time for demetallation (preparation of pheophytin a)	Hexane, 1 N HCl, 4 h	Hexane, 2 N HCl, 2 h
Base used for Ce6 synthesis	1 M NaOH	13% ethanolic solution of KOH
Time duration for Ce6 synthesis	12 h	10 min
Ce6 purification	pH change, centrifugation, and filtration	pH change, filtration
Total time	5 days	3 days
Yield % and Purity %	<1% of Spirulina mass and <97%	<1% of Spirulina mass and <94%

**Table 2:** The forward primer (FP) and reverse primer (RF) sequences used for qPCR.

Oligo Name	Oligo Sequences
TNF-α FP	AGC CCA TGT AGC AAA CC
TNF-α RP	GGA AGA CCC CTC CCA GAT AG
IFN-γ FP	TTC AGC TCT GCA TCG TTT TG
IFN-γ RP	CAT GTA TTG CTT TGC GTT GG
IL-2 FP	TGC AAC TCC TGT CTT GCA GC
IL-2 RP	ATG GTT GCT GTC TCA TCA GC

## Data Availability

All the data are contained within the article and Supplementary Materials.

## Supplementary Materials

The following supporting information can be downloaded at: https://www.mdpi.com/article/10.3390/ijms24043901/s1.
