# Supplementary material for: Efficient Synthesis of Chlorin e6 and Its Potential Photodynamic Immunotherapy in Mouse Melanoma by the Abscopal Effect
Source: Int J Mol Sci. 2023 Feb 15;24(4):3901. doi: 10.3390/ijms24043901 (PMC9963834; doi:10.3390/ijms24043901)
Supplement: Supplementary file 1 [file ijms-24-03901-s001.zip › ijms-2112568-supplementary.pdf]

Supplementary Information

Efficient Synthesis of Chlorin e6 and its Potential Photodynamic Immunotherapy in Mouse Melanoma by the Abscopal Effect

Rajeev Shrestha [†], Shyam Kumar Mallik [†], Junmo Lim, Pallavi Gurung, Til Bahadur Thapa Magar and Yong-Wan Kim [*]

Dongsung Cancer Center, Dongsung Biopharmaceutical, Daegu 41061, Republic of Korea
* Correspondence: thomas06@hanmail.net
† These authors contributed equally to this work.

Table of Contents

S1. HPLC analysis of chlorophyll a by method 1

S2. HPLC analysis of pheophytin a by method 1

S3. $^1H_1$ NMR spectra of Ce6 by method 1

S4. HPLC analysis of chlorophyll a by method 2

S5. HPLC analysis of pheophytin a by method 2

S6. $^1H_1$ NMR spectra of Ce6 by method 2

S7. Pharmacokinetics of Ce6

S1. HPLC analysis of chlorophyll a by method 1

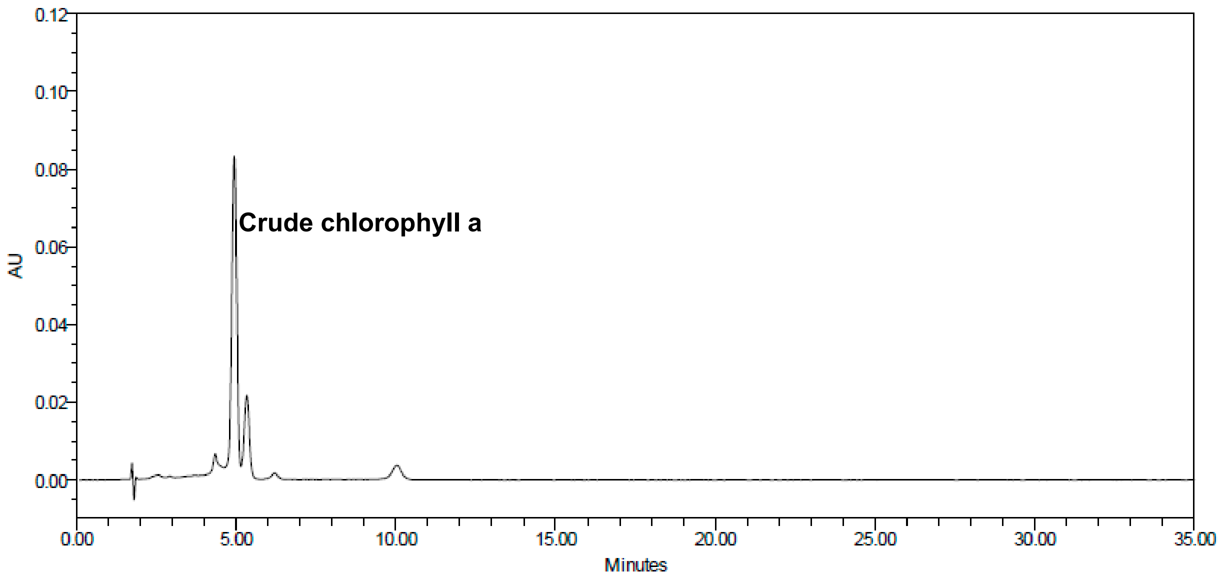

Figure S1. HPLC chromatogram of Chlorophyll a (method 1) at wavelength 430 nm.

S2. HPLC analysis of pheophytin a by method 1

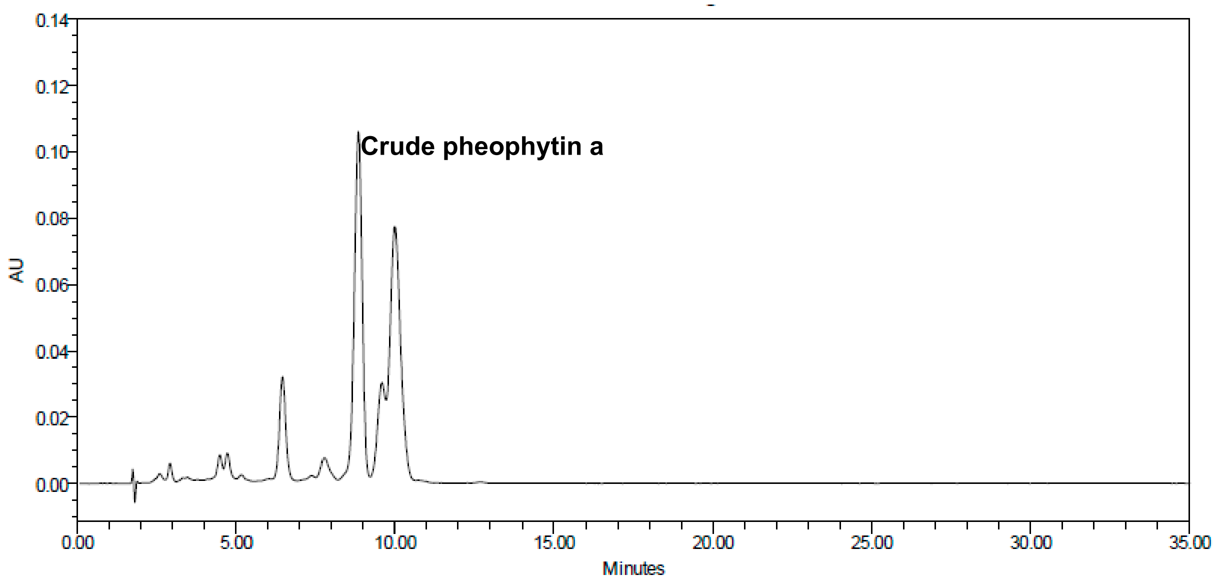

Figure S2. HPLC chromatogram of pheophytin a (method 1) at wavelength 430 nm.

S3. $^1H_1$ NMR spectra of Ce6 by method 1

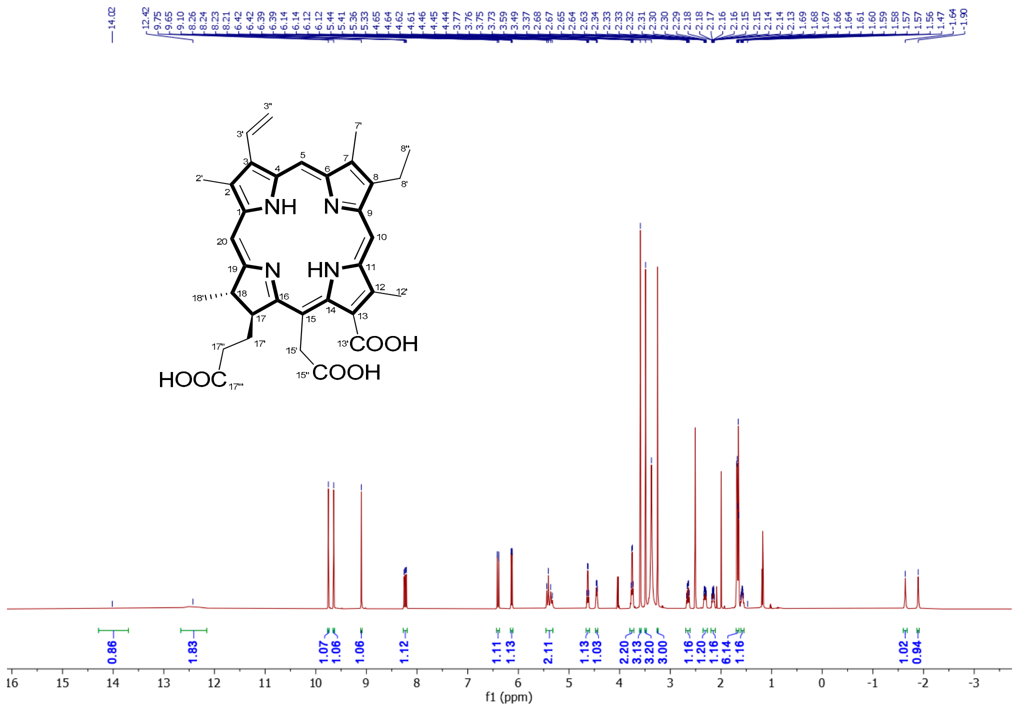

Figure S3. ^1H NMR of Ce6 (method 1). NMR was analysed in solvent DMSO-d_6.

S4. HPLC analysis of chlorophyll a by method 2

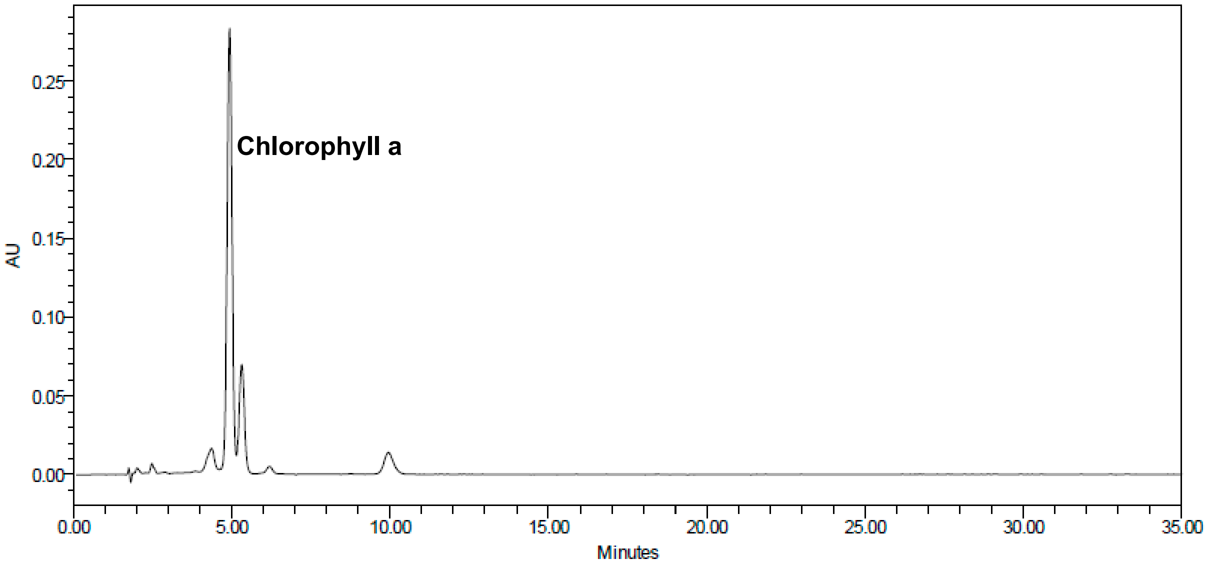

Figure S4. HPLC chromatogram of Chlorophyll a (method 2) at wavelength 430 nm.

S5. HPLC analysis of pheophytin a by method 2

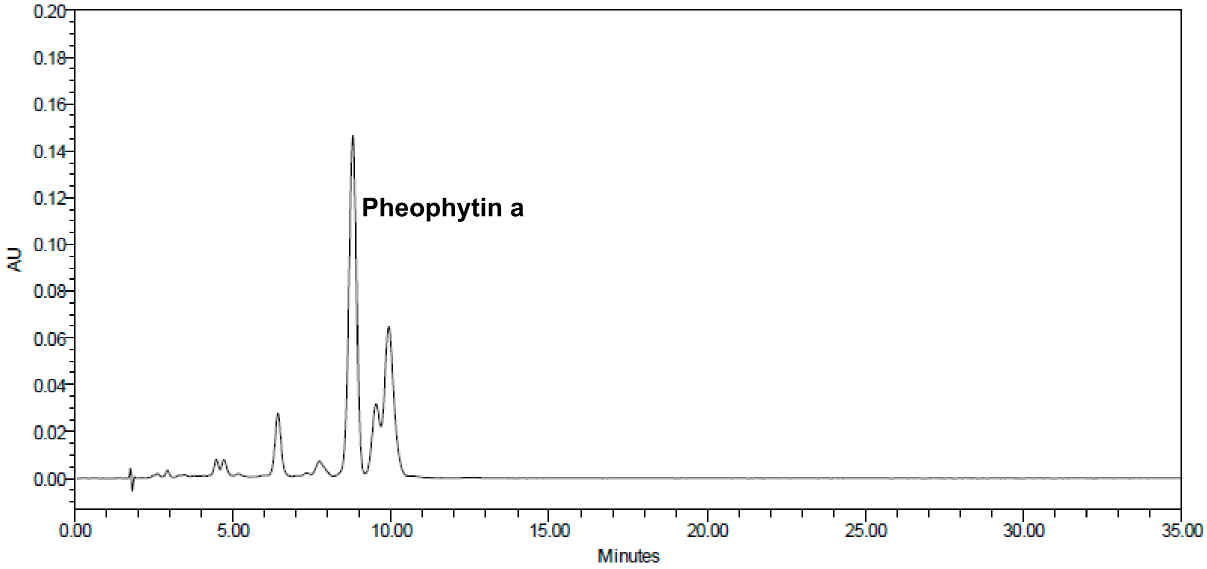

Figure S5. HPLC chromatogram of pheophytin a (method 2) at wavelength 430 nm.

S6. $^{1}H_{1}$ NMR spectra of Ce6 by method 2

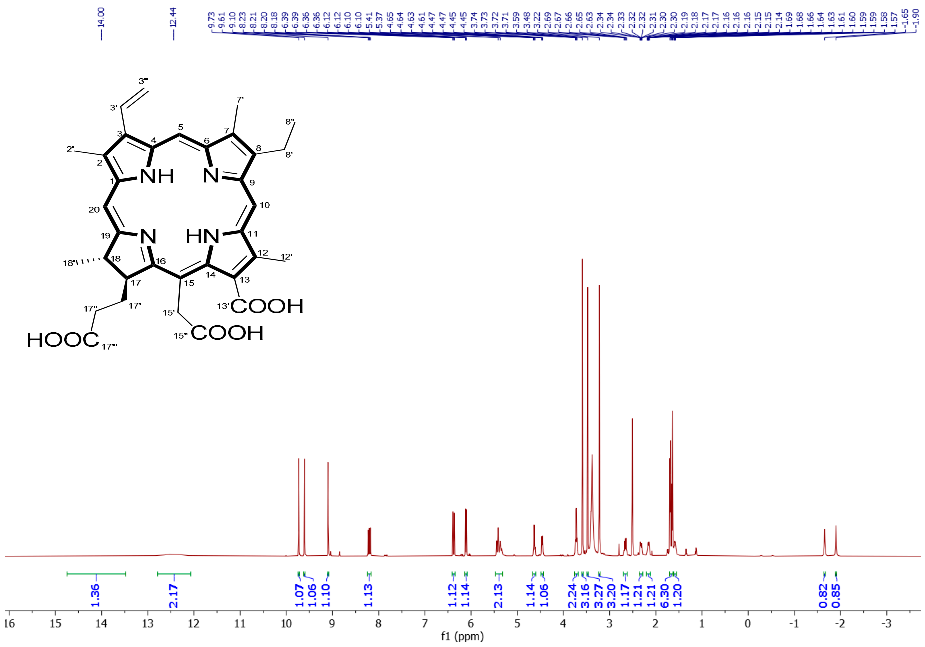

Figure S6. ^{1}H NMR of Ce6 (method 2). NMR was analysed in solvent DMSO-d_6.

S7. Pharmacokinetics of Ce6

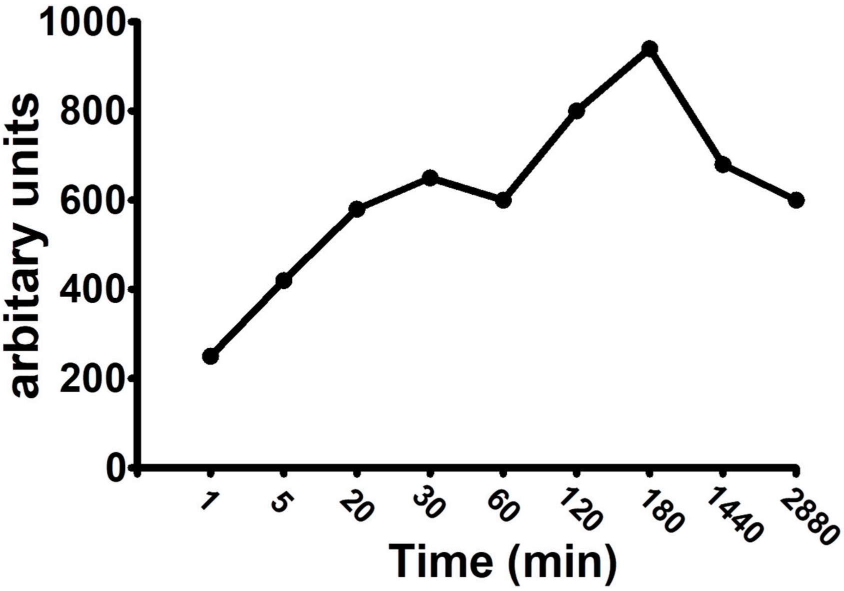

Figure S7. Changes in intravital fluorescence intensity with the time (min) in normal tissue (femur) of rat after intravenous administration of Ce6:PVP (1:1) at dose of 1 mg/kg (n = 3).

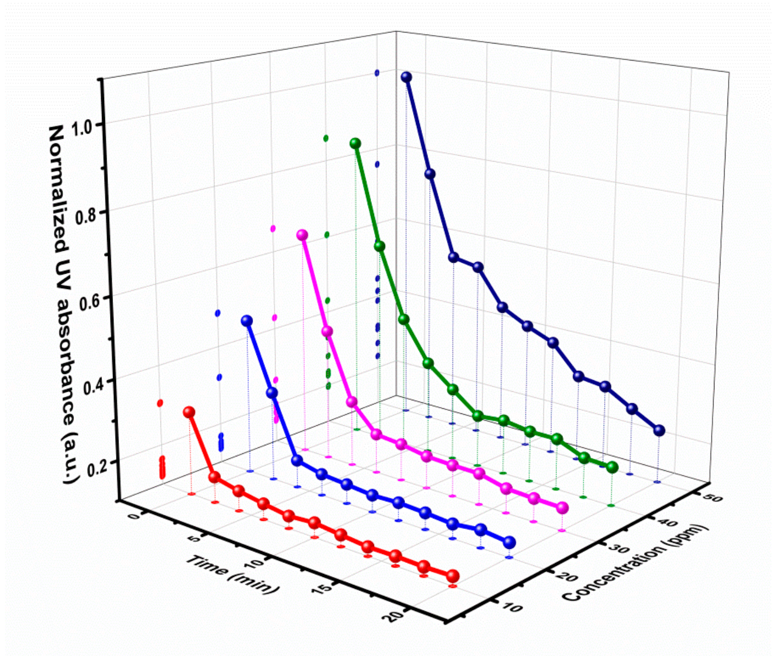

Figure S8. The decay of Ce6 (10-50 ppm) after light irradiation (660 nm) in 0 to 20 min. The normalized UV absorbance was measured in 662 nm.
